# 1583. Optimal timing of antiretroviral therapy initiation in AIDS-associated Toxoplasma encephalitis: a prospective observational multicenter study in China

**DOI:** 10.1093/ofid/ofad500.1418

**Published:** 2023-11-27

**Authors:** yaokai Chen, Yao Li

**Affiliations:** Chongqing Public Health Medical Center, chongqing, Chongqing, China; Chongqing Public Health Medical Center, chongqing, Chongqing, China

## Abstract

**Background:**

Toxoplasma encephalitis (TE) is the most frequent cause of expansive brain lesions among acquired immunodeficiency syndrome (AIDS) patients; however, the optimal timing of antiretroviral therapy (ART) initiation in these patients remains controversial.

**Methods:**

This was a multicenter prospective observational study, and eligible patients were recruited from eleven treatment centers in China.

**Results:**

**In total**, **87 patients were included**, and 38 of them were assigned to the Early ART group (initiating ART within 2 weeks after anti-Toxoplasma treatment initiation), **while the remaining 49 patients were allocated to receive deferred ART (initiating ART at least 2 weeks after anti-Toxoplasma treatment initiation). Our results indicated that the incidence of immune reconstitution inflammatory syndrome (IRIS) (2.6% vs. 0**, **p=0.437) and the number of death events (1 vs. 5**, p=0.225) were not significantly different between the two groups at Week 48. The timing of ART initiation was also found to not significantly contribute to human immunodeficiency virus (HIV) viral load control. The difference in the number of patients who maintained an undetectable HIV viral load of <50 copies/mL in each of the two groups of patients was calculated to not be statistically significant at Week 24 (8 vs. 3, **p=0.142) and Week 48 (7 vs. 7**, **p=1.000). Meanwhile**, median CD4+ T-cell counts were also observed not to reach statistical significance between the two groups, **both at Week 24 (155 vs. 91**, **p=0.837) and Week 48 (181 vs. 96**, **p=0.219).**

**Conclusion:**

In our study, early ART initiation was observed to not confer statistically significant differences in the incidence of IRIS, mortality, and HIV virological and immunological outcomes, when compared to deferred ART initiation.

**Disclosures:**

**All Authors**: No reported disclosures

